# Noncovalent Immobilization of *Yarrowia lipolytica* Lipase on Dendritic-Like Amino Acid-Functionalized Silica Nanoparticles

**DOI:** 10.3390/biom9090502

**Published:** 2019-09-18

**Authors:** Zahra Fathi, Esmail Doustkhah, Golamhossein Ebrahimipour, Farshad Darvishi

**Affiliations:** 1 Department of Microbiology, Faculty of Biological Technology, Shahid Beheshti University, Tehran 19839-63113, Iran; 2 Young Researchers and Elite Club, Maragheh Branch Islamic Azad University, Maragheh 55197-47591, Iran; 3 Microbial Biotechnology and Bioprocess Engineering (MBBE) Group, Department of Microbiology, Faculty of Science, University of Maragheh, Maragheh 55181-83111, Iran

**Keywords:** *Yarrowia lipolytica*, lipase, biocatalysis, immobilization, dendrimer

## Abstract

Immobilization of enzymes is a promising approach for the cost-effective application of enzymes. Among others, noncovalent but unleachable approaches for immobilization are one of the most favorable and crucial approaches. Herein, silica nanoparticles are modified by (3-aminopropyl)triethoxysilane (APTES) to generate amino-functionalized silica nanoparticles. Then, the amine functionalities are converted to bifunctional amino acid via post-modification that has zwitterionic properties. This nanostructure with the new functional theme is employed to immobilize *Yarrowia lipolytica* lipase at room temperature with no further post-modification or cross-linking. This immobilization method is further compared with the metal chelate-based immobilization approach on the same support. The biocatalytic activity of the immobilized lipase is examined under various conditions. The encapsulation of lipase through amino acid-functionalized silica nanoparticles exhibited enhanced stability for the immobilized lipase at higher temperatures and unneutral pHs.

## 1. Introduction

Lipases have attracted a high domain of biocatalytic processes. This capability of lipases has been dominated by many industries such as biodiesel production [1,2], the food industry [3] and synthetic pharmaceuticals [4,5]. Lipases can be produced as extra/intracellular and cell-bonded routes by organisms. Microbial production of lipases plays an important role in industry due to the capability of a large scale and lower costs of production than other organisms.

*Yarrowia lipolytica* is one of the most important yeast species that produces several intracellular, cell-bound, and extracellular lipases. All lipases obtained from this yeast are classified within biosafety class 1 and they can be recognized as an excellent option in food and medicinal industries. Among several lipases of this yeast, *Y. lipolytica* extracellular lipase (YLIP2) has significant importance since it has high biocatalytic activity and acts actively in ester and biodiesel biocatalysis as well as in other applications [6,7,8].

Lipases are meritorious due to their good stability in a broad range of pH levels and temperatures. Moreover, they need no activation step by the addition of a cofactor, tolerate harsh conditions in industrial processes, can be genetically manipulated with ease, consume lower energy, and produces less waste compared to other biochemical processes [9]. However, there are some drawbacks in lipase utilization as the biocatalyst in the industry, such as the high cost of lipase and low stability during the processing. To overcome these drawbacks, lipases immobilization is an inevitable strategy to make them economically suitable for industrial applications through convenient recovery and recycling from the reaction mixture beside increasing the lipase stability [10,11].

Among all the available enzyme immobilization techniques, the tendency toward metal-free approaches has been raised up to reach green and non-toxic protocols, which could be compatible to the environment with minimum release of pollutants [12,13,14]. Hybrid silica nanostructures such as the heterogeneous and metal-free supports certainly provide green credentials for catalytic processes [15,16]. This approach can minimize the amount of generated waste and side-products out of chemical reactions.

Silica nanostructures have a high versatility in nanoarchitecture through many common approaches [17,18]. Hybridization of these materials with organosilicon-based compounds is a unique strategy in making them more efficient for catalysis and biocatalysis [18]. For instance, immobilizing enzymes on silica needs this hybridization. However, many methods of enzyme immobilization rely on cross-linking and covalent immobilization [19]. In most cases, these methods deal with drawbacks such as inactivation of the enzyme by occupying the active sites of the enzyme and affecting the rotational mobility [20] or increasing the steps of preparation which make the process unsuitable for an efficient (photo)(bio)catalytic process. In contrast, electrostatic immobilization through ionic species is superior and preferable since none of the mentioned drawbacks may exist in the electrostatic immobilization [21]. The ionic liquid is a typical and most common type of agents for electrostatic immobilization which is widely studied [22,23] while zwitterionic type functionalities have attracted attention very recently [24]. Thus, we examined the capability of these species in lipase immobilization on amino acids. Among various nanomaterials, silica nanostructures are attractive and popular nanomaterials thanks to their high stability in various conditions and high surface area. Moreover, their nanoarchitecture is likely to be functionalized by a wide range of organic precursors e.g., organosiloxanes. Therefore, they are well-employed in a vast range of applications including catalysis, biocatalysis, electrocatalysis, biosensors, metal adsorption, medical cases, and drug delivery [25,26,27,28,29].

In this work, we have employed hybrid silica nanoparticles bearing dendritic-like amino acids for electrostatic immobilization of *Yarrowia lipolytica* lipase (LIP2) at room temperature. Then, we investigated the stability and performance of the lipase within the matrix of support compared to its free form. We found a recyclable, synergistic, and metal-free approach for biocatalytic processes.

## 2. Materials and Methods

### 2.1. Materials and Characterization

Extracellular lipase (YLIP2) was produced from *Y. lipolytica* U6 according to the reported method [30]. In this work, *p*-nitrophenyl laurate (PNPL, Sigma-Aldrich Co.) was used as a hydrolysable substrate for lipase. Hydrolysis of the substrate was monitored by releasing colorful *p*-nitrophenol (PNP) using UV-Vis spectroscopy (UV-1800 Shimadzu). Scanning electron microscopy (SEM, Zeiss-DSM 960A microscope) was used to perform the elemental mapping and studying the morphology changes. 

### 2.2. Lipase Production and Detection

YLIP2 was produced according to previously reported literature [31]. Briefly, *Y. lipolytica* U6 strain was activated by yeast extract–peptone-dextrose (YPD, 20 mL) media (dextrose: 20 g, peptone: 20 g, and yeast extract: 10 g) overnight and inoculated in 200 mL YPD in a flask (initial OD_600_ of 0.05). The cell-free culture supernatant was obtained after 48-h incubation at 29 °C by centrifugation (5000 rpm for 5 min in 4 °C). Finally, supernatant including extracellular lipases was freeze-dried at 20 °C with 0.5% of maltodextrin to obtain concentrated lipase powder. Lipase activity assay was assayed by using PNPL as a substrate by the procedure in literature [32]. The supernatant absorbance at 410 nm was monitored after incubating at 37 °C for 10 min via UV-Vis spectroscopy. Lipase activity unit was defined in terms of µmol of released PNP from the substrate per min [32].

### 2.3. Synthesis of SiO_2_/AA

Silica nanoparticles with particle size of 10–20 nm were purchased from Sigma-Aldrich company (nanoparticles, nonporous, molecular weight of 60.08 g/mol) and used for modifications. In a general procedure, silica nanoparticles powder was first functionalized by 3-aminopropyltriethysilane (APTES) and subsequently, the amino-functionalized silica nanoparticles (SiO_2_-NH_2_) were reacted with sodium chloroacetate. Specifically, 1 g of silica nanoparticles were dispersed in toluene (15 mL) for 30 min at room temperature and then to the dispersion, APTES (10 mmol, 2.25 mL) was added to the reaction under same conditions and the temperature was raised to 90 °C and stir for 48 h. Finally, the product was collected by filtration on filter paper, washed with EtOH and dried in an oven at 80 °C for 2 h. Afterward, the as-obtained SiO_2_/PrNH_2_ was dispersed by ultrasonic irradiation in ethanol (20 mL) containing sodium chloroacetate (30 mmol, 3.5 g), triethylamine (TEA, 10 mL) and EtOH (15 mL) and the reaction was kept at reflux conditions for 48 h. Then, the solid was centrifuged and washed with pure EtOH for several times and dried at 80 °C for 2 h [33]. Finally, the material was dispersed in water and its pH was adjusted to 6.5 by diluted HCl (10^−4^ M) and collected by centrifugation and dried at 100 °C. The final product was named SiO_2_/AA. 

### 2.4. Metal Ion Chelation on SiO_2_/AA

For the chelation, 1 g of SiO_2_/AA was dispersed in aqueous solution of metal acetates (1 mM) at room temperature and stirred for 12 h. Then, the reaction was stopped and a solid was recovered by centrifugation and washing with distilled water for three times. The final product was dried under vacuum at room temperature. 

### 2.5. Lipase Immobilization

Lipase immobilization on SiO_2_/AA was performed based on the following method: initially, different amounts of the support (5, 10, 15, 20 and 25 mg) were added to phosphate buffer solution (100 mM, 1 mL, pH = 7) containing free lipase (10 mg/mL) and incubated at 37 °C for 12 h, under magnetic stirring mode. Then, the obtained products were collected by centrifugation and washed with the same buffer, obtaining SiO_2_/AA@YLIP2. The residual enzyme in the supernatant was measured by protein assay (Bradford method) to estimate the amount of the immobilized lipase. 

The thermal stability of immobilized enzyme was investigated by incubating at 25, 30, 35, 40, 45, and 50 °C for 4 h in a water bath. The pH stability of immobilized enzyme was investigated by incubating the immobilized enzyme or lipase at different pH (4, 5, 6, 7, 8 and 9) for 4 h at room temperature. The effect of temperature for hydrolyzing activity was investigated by incubating at 25 °C, 30 °C, 40 °C, and 50 °C for 10 min in a water bath. The effect of pH for hydrolyzing activity of immobilized enzyme was investigated by incubating the immobilized enzyme at different pH (4, 5, 6, 7, 8 and 9) for 10 min in 37 °C at pH = 7. The experiments were conducted in triplicates.

### 2.6. Reusability of Immobilized Lipase

For studying the reusability of the immobilized lipase, the immobilized lipase was recovered by centrifugation after hydrolysis reaction at 5000 rpm for 3 min and washed twice with phosphate buffer (pH = 7) for three times. Then, the obtained solid was reused for the next cycle of the hydrolysis reaction. The efficiency of immobilized lipase examined for five consecutive cycles under mentioned conditions. All sampling was performed in triplicate.

## 3. Results

**Figure 1:**
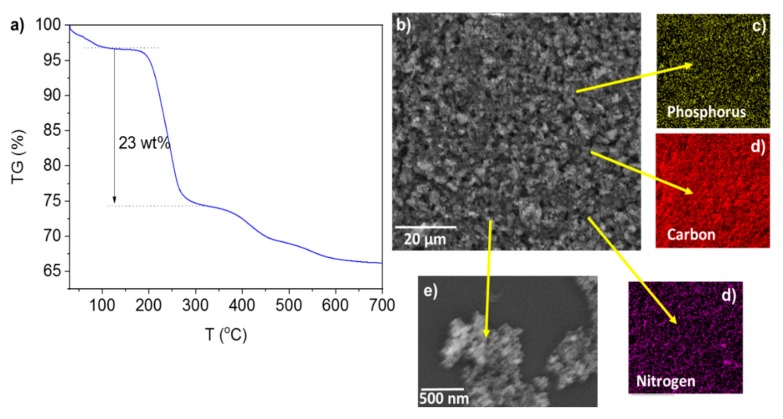
(**a**) TGA profile of SiO_2_/AA, (**b**)low-magnified SEM image of SiO_2_/AA@YLIP2 and its SEM mapping showing, (**c**–**e**) related elements, and (**f**) high-magnified SEM image of SiO2/AA@YLIP2.

Thermal gravimetric analysis (TGA) was performed under O_2_ atmosphere with heat ramp of 5 °C/min in a Pt pan (Model of TGA: TA Q600, USA). According to obtained profile, there is a weight loss (~23 wt%) in thermal TG profile of SiO_2_/AA in air ranging from 200–300 °C (Figure 1a). Elemental SEM-mapping of SiO_2_/AA@YLIP2 was performed (model TE-SCAN, on 5 kV) to confirm the immobilization of lipase (LIP2) on the surface of SiO_2_/AA. Moreover, the high magnification SEM image of SiO_2_/AA@YLIP2 shows the morphology of the silica nanoparticles carrying lipase (Figure 1).

### 3.1. The Hydrolytic Activity of Immobilized Lipase

**Figure 2:**
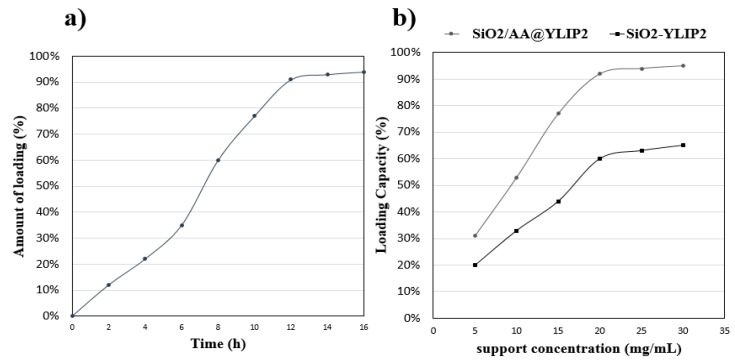
(**a**) Effect of incubation time on the adsorption of lipase onto SiO_2_/AA (Conditions: PNPL (1 mM, 900 µL), SiO_2_/AA (25 mg/mL), at 37 °C). (**b**) Comparison of lipase loading capacity between SiO_2_/AA and SiO2 (Conditions: YLIP2 powder (10 mg/mL) (100 mM, 900 µL), support (30 mg/mL, 100 µL) during 12 h at 37 °C. 100% activity = 4600 U/g).

The final activity of the concentrated lipase powder in terms of PNPL hydrolysis as the substrate was obtained ~5000 U/g. Besides, the amount of YLIP2 immobilization on SiO_2_/AA was found: ~4600 U/g. By comparing the activity of the immobilized lipase and free lipase per g of catalyst, the activity of immobilized lipase was significantly maintained during the immobilization step. This observation shows that our proposed matrix and nanomaterial has high adaptability with extracted LIP2 and does not deactivate the active sites of the enzyme. Since our support has organocatalytic activity, as we have discussed before [34], we found that it (SiO_2_/AA) also exhibits a similar organocatalytic activity in the hydrolysis of PNPL (15% hydrolysis activity). Therefore, the support not only immobilizes the lipase but also has a co-catalytic effect on the hydrolysis of the PNPL. Aside from the organocatalytic activity of the support, the 92% of lipase’s activity (~4600 U/g) was retained after immobilization compared to the initial activity of the lipase (5000 U/g, free concentrated lipase activity). Since the pure silica surface is somehow capable of adsorbing lipase onto its unmodified surface thanks to the silanol-rich surface, the present silica nanoparticles in this project were nakedly tested in the immobilization of lipase. The results showed that this type of silica nanoparticles can partially immobilize lipase and its activity in the PNPL hydrolysis is around ~63%, which agrees with previous reports [20]. Loading capacity of the support was measured by monitoring the residual lipase in the supernatant of the immobilization dispersion using the Bradford method. As indicated in Figure 2, the first 10 h had the highest rate of immobilization and afterward, the rate of immobilization decreases to zero within 16 h (Figure 2a). 

**Figure 3:**
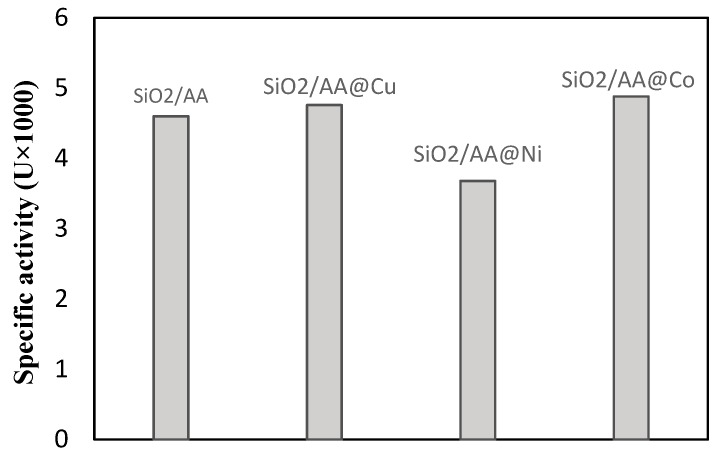
Chelating effect of the transition metals on the specific activity of the immobilized LIP2 on SiO_2_/AA (in all cases, the enzyme is immobilized on the surface of SiO_2_/AA).

We supported a series of transition metal ions on the surface of SiO_2_/AA. We immobilized YLIP2 on every individual support which contains a specific metal ion to observe the effect of chelation on the immobilization and activity of lipase (Figure 3).

**Figure 4:**
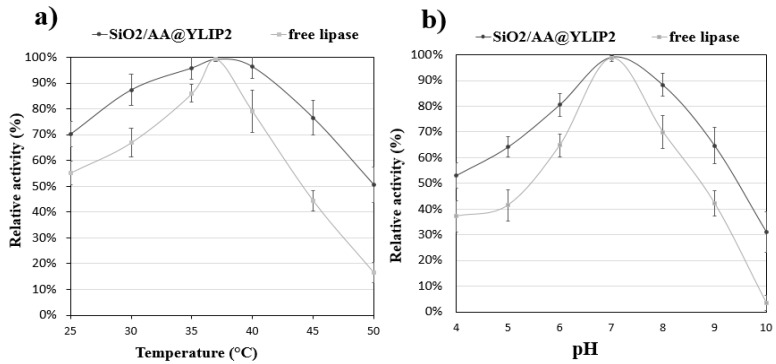
Performance of free lipase and SiO_2_/AA@YLIP2 at (**a**) different temperatures and (**b**) different pH (Relative activities were calculated with reference to the activity of immobilized lipase at 37 °C and pH = 7. 100% activity = 4600 U/g).

We examined the effect of temperature on the performance of both immobilized and free lipase and compared to those of normal and neutral conditions. The activity of SiO_2_/AA@YLIP2 in PNPL hydrolysis was monitored under the different temperatures (e.g., 25, 30, 40 and 50 °C) with reference to 37 °C (Figure 4a). Then, the effect of pH on the performance of immobilized lipase was monitored under different pHs (pH 4, 5, 6, 7, 8, and 9) and compared to the free lipase with reference to its neutral conditions (Figure 4b). The optimal pH for both free lipase and SiO_2_/AA@YLIP2 was found at pH = 7 while under acidic and alkaline conditions, the performance of the lipase in both conditions has a relative decrease. 

**Figure 5:**
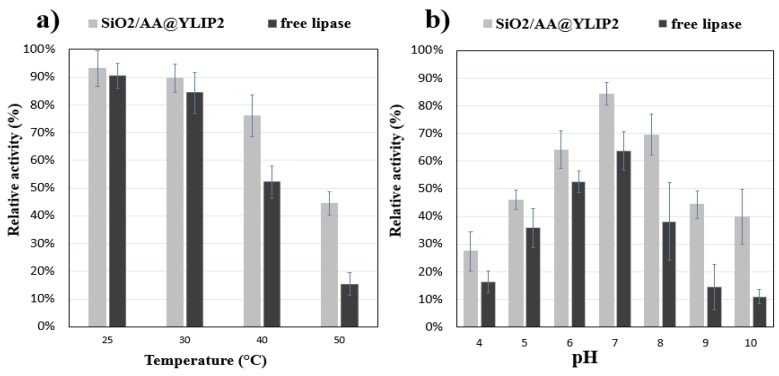
(**a**) Comparing the relative activity of SiO_2_/AA@YLIP2 and free lipase at pH = 7 after exposing the lipases in different pH for 4 h (Conditions: PNPL (10 mg/mL, 1 mg), lipase (10 mg) in case of supported lipase (30 mg) at 37 °C in 4 h); (**b**) thermal stability of free lipase and SiO_2_/AA@YLIP2 by exposing them in different temperatures for 4 h (Conditions: PNPL (10 mg/mL, 1 mg), lipase (10 mg) or supported lipase (30 mg) at pH = 7 in 10 min; relative activities were calculated with reference to the activity of immobilized lipase at 37 °C and pH = 7. 100% activity = 4600 U/g).

We further studied the stability of the immobilized lipase rather to its corresponding free lipase. We kept the lipase (both free and immobilized lipase) at various temperatures (e.g., 25 °C, 30 °C, 40 °C, and 50 °C) and pHs ranging in 4–9 for 4 h (Figure 5). Then, they were exposed to the substrate, PNPL in buffer solution pH = 7, and the activity of lipase in each condition was compared to its fresh lipase which has undergone no temperature or pH shock. This investigation showed that SiO_2_/AA act as protector to keep the lipase more stable under harsh condition (e.g., at higher temperatures, alkaline or acidic conditions). After 4 h of heating at 50 °C, the free enzyme only retained 13% of its activity while in the case of SiO_2_/AA@YLIP2, it retains more than 40% of its activity. 

### 3.2. Reusability of SiO_2_/AA@YLIP2

**Figure 6:**
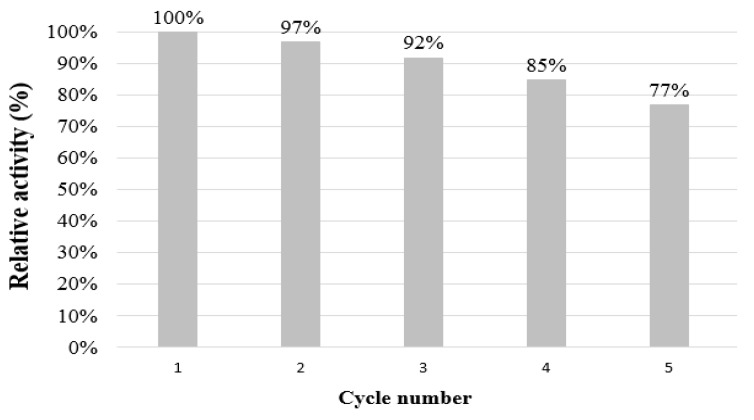
Activities of recovered SiO_2_/AA@YLIP2 for five consecutive cycles (100% activity = 4600 U/g).

Reusability of YLIP2 on SiO_2_/AA was investigated to understand the efficacy of the support in preserving the lipase on its surface. determined by residue activity of enzyme solution for several cycles as the most important index immobilizations of the enzyme. The hydrolytic activity gradually decreased to 77% of the initial activity after five consecutive runs which in turn is unique in reusability of the lipase in electrostatic approach (Figure 6). 

## 4. Discussion

**Scheme 1:**
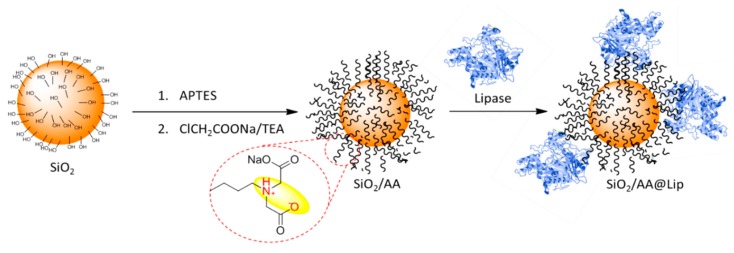
A glimpse at the post-modification of silica nanoparticles and immobilization step lipase on SiO_2_/AA.

Synthesis of SiO2/AA@YLIP2 was performed starting from post-modification of uniform silica nanoparticles (~20 nm) with APTES and then with chloroacetate to produce amino acid functionalities on the surface. Since there is a bifunctionality, the surface has a zwitterionic feature and can be a suitable platform for immobilization of lipase. Therefore, it was used for immobilization of YLIP2 through noncovalent approach (Scheme 1). 

We confirmed the presence of organic motifs which are functionalized and post-modified onto the silica nanoparticles through a sharp weight loss in 200–300 °C clearly shows that SiO_2_/AA is composed of organic groups. Thus, we confirmed the presence of corresponding elements, specifically phosphorus, nitrogen, and carbon (Figure 1) through this analysis. The presence of phosphorus atom indicates the lipase is successfully immobilized on the surface since the phosphorus elements refer to lipase. Moreover, the high-magnification SEM image of SiO_2_/AA@YLIP2 shows that all post-modification steps and immobilization process have not distorted the parental morphology of the silica nanoparticles (Figure 1).

According to the results, the lipase immobilization capacity was raised from 60 to 92% after post-modification of the silica nanoparticles through amino acid groups. There was a very slight increase in lipase activity by increasing the ratio of the lipase to support amount that can be attributed to the fact that the covalent binding of the enzyme to the support slightly blocks the active sites of the enzyme. 

Since there are many reported cases about the immobilization of lipase by chelating to an existing transition metal (e.g., Cu^2+^) so that the efficiency of immobilization can be increased [35]. Here, we supported a series of transition metal ions on the surface of SiO_2_/AA. We immobilized YLIP2 on every individual support which contains a specific metal ion. This examination indicated that Ni^2+^ has a negative effect on the activity of LIP2 while other ions have only a trivial positive effect on boosting the specific activity. Therefore, the LIP2 did not need any chelating agent to boost the enzyme activity (Figure 3).

The superior hydrolysis performance of SiO_2_/AA@YLIP2 than free lipase at the higher temperatures and relatively at lower temperatures shows that immobilizing the lipase on SiO_2_/AA makes it more resistant to any thermal changes (Figure 4a). The extent of this decrease was critically dependent upon the presence of SiO_2_/AA encapsulating the lipase. Therefore, when the lipase was in immobilized form, it showed a higher resistance rather to its free form. This study shows that the lipase in this immobilized form is more resistant to the media’s pH changes. Also, temperature rising exhibits a lower decrease in the activity of the lipase when it is immobilized onto SiO_2_/AA, implying more tolerance to denaturation rather than free state (Figure 5). 

The results of recyclability (Figure 6) shows that the electrostatic interaction in this system is somehow a strong interaction that retains the major fraction of lipase on the surface. Referring to the previous section about the stability of the immobilized at a higher temperature, it can be noticed that a fraction of immobilized lipase undergoes leaching while another fraction undergoes lipase deactivation during each run and recovery process.

## 5. Conclusions

In summary, here we introduced a new efficient support with synergic effect for immobilization of YLIP2 through the electrostatic approach. SiO_2_/AA as a heterogeneous organocatalyst can both immobilize the LIP2 and synergize the biocatalytic activity of the immobilize LIP2. Furthermore, it acted as a protector in retaining the 3D conformation of the lipase at higher temperatures and unnatural conditions. Immobilization process had an insignificant negative effect in decreasing the activity of the lipase compared to the parent or free lipase. Existence of Ni^2+^ ion on the surface of support causes a decrease in the activity of the LIP, which is likely owing to the mal-effect on the active conformation of the LIP2. In contrast, Cu^2+^ and Co^2+^ didn’t have a negative effect on the specific activity of the LIP2. SiO_2_/AA@YLIP2 was proved to be a recyclable biocatalyst at least for five consecutive runs with a small decrease in the activity in each run. Moreover, the immobilized lipase was more active in the acidic and alkaline and even higher temperatures. This support showed good sustainability and biocompatibility to the lipase during all processes while no chelating agent or covalently linking agent was used for immobilization onto the surface. This support may be a good candidate for other types of enzymes for immobilization at room temperature and ambient conditions.
